# Investigating Frontline Nurse Stress: Perceptions of Job Demands, Organizational Support, and Social Support During the Current COVID-19 Pandemic

**DOI:** 10.3389/fpubh.2022.839600

**Published:** 2022-05-25

**Authors:** Haneen Ali, Yasin Fatemi, Duha Ali, Mohammad Hamasha, Sa'd Hamasha

**Affiliations:** ^1^Department of Industrial and Systems Engineering and Health Services Administration Program, Auburn University, Auburn, AL, United States; ^2^Department of Industrial and Systems Engineering, Auburn University, Auburn, AL, United States; ^3^Department of Industrial Engineering, Faculty of Engineering, The Hashemite University, Zarqa, Jordan

**Keywords:** nursing staff, COVID-19, supervisor support, social support, coping strategies

## Abstract

**Background:**

While frontline nurses employ coping alternatives to help deal with occupational stress resulting from unprecedented challenges during the COVID-19 pandemic, their access to necessary resources is unclear.

**Objective:**

This study aims to explore nurses' mental health in Alabama hospitals during the COVID-19 outbreak and investigate the impact of organizational and community support on nurse stressor levels, physio-psychosocial responses, and coping strategies employed.

**Methods:**

A cross-sectional survey was developed to bridge our understanding of stress, support, and coping mechanisms and distributed to nurses working with COVID-19-infected patients in hospital settings in Alabama. A total of 232 frontline nurses responded to 79 items in four domains (stressors, physio-psychosocial symptoms, coping, and support) between May 6, 2020, and June 30, 2020. A two-way ANOVA, regression analysis, and mediation of effects were used to analyze the data.

**Results:**

This study found that both social support and use of coping strategies contributed to the reduction of physio-psychosocial symptoms. Differences were found in how older frontline nurses perceived the efficacy of social support and certain coping strategies. This study provides further evidence of the importance of organizational support in addressing the harmful physio-psychosocial symptoms experienced by nurses.

## Contribution of the Paper

### What Is Already Known

- The COVID-19 pandemic has impacted the health and psychological wellbeing of nursing staff working with infected patients.
- While additional support is crucial during pandemics, little is known about the impact of organizational resources and supervisor and community support on nurses' stress levels, physio-psychosocial responses, and coping strategies.

### What This Paper Adds

- An understanding of nursing staff stressors, resulting physio-psychosocial symptoms, and coping mechanisms employed.
- An understanding of the impact of social support and coping support on the reduction of physio-psychosocial symptoms.
- A better understanding of how the generational context affects nurses' perceptions of various approaches and levels of support.

## Background

### Frontline Nurse Challenges

As of August 21, 2021, the number of COVID-19 cases in the United States had reached over 37 million confirmed cases and over 625,000 deaths, with more than 788,000 healthcare professionals infected, and more than 3,000 dead (1). The COVID-19 pandemic has significantly impacted healthcare professionals' psychological health (2–4). Evidence suggests that during the pandemic, nurses struggle with psychological problems and suffer adverse mental and emotional symptoms, such as depression and stress (2, 5, 6). Previous studies reported sources of stressors and the emotions of nursing staff resulting from the pandemic (2, 7–9), others reported the psychosocial impact and coping strategies employed by nurses (2, 6, 9, 10). Other studies reported the effects of work stress on nursing staff burnout (8, 11–15). However, only few studies investigated the effects of organizational and community support in addressing the adverse psychological effects of COVID-19, and its relationship to the coping strategies deployed by the nursing staff (16–19).

The pandemic in Alabama provides frontline nurses with challenges, not only due to a greater workload from infections, but also additional adverse psychological effects that local hospitals may be ill equipped to address (2, 20, 21). Failure to address these problems could negatively impact healthcare workers and cause short- and long-term psychological injuries (22, 23).

Support from workplaces, friends, family, and colleagues could balance and sustain this emotional stress and provide nursing staff with coping mechanisms that safeguard their wellbeing and mental health (6, 24). Organizational resources are designed to help reduce uncertainty caused by shortages, such as personal protective equipment (PPE), ventilators, medical countermeasures, and health care providers (2, 20, 25). Organizational support is meant to reducing mental and psychological health deterioration resulting from the pandemic. Organizational support can involve providing mental health resources and a clear flow of information, which can alleviate uncertainty and fear (26, 27). Studies suggest that nurses' fears and anxiety symptoms could be addressed through strong, clear communication with nursing staff and regular updates on the COVID-19 outbreak (2, 20, 25). Studies have also found that nurses tend to feel more emotionally exhausted when they do not receive adequate supervisory support (2, 20, 28). Supervisor support plays a crucial role in reducing frontline nurse stress resulting from working in a hectic environment, which may lead to emotional exhaustion and can affect their health and wellbeing (2, 20, 29).

Social support from family and friends can reduce emotional exhaustion and stress and protect against physio-psychosocial symptoms (9, 30). It has been reported that social relationships and support from friends have a mediating effect on stress and physio-psychosocial symptoms, and can help mitigate the stress and anxiety from working as a frontline nurse (2, 5, 20, 24, 30, 31). Social support is reported to help nurses by allowing them to relate personal experiences to each other (2). This interpersonal and self-affirming aspect of social support may help explain how nurses in Alabama use transference as a coping strategy, despite never using psychological counseling (2). Organizational and social support could reduce occupational stress and improve psychological wellbeing by providing a protective layer against anxiety, stress, and depression, and impact the coping mechanisms employed by nursing staff (5, 20, 30).

#### Stress-Symptom-Support-Coping Framework

**Figure 1:**
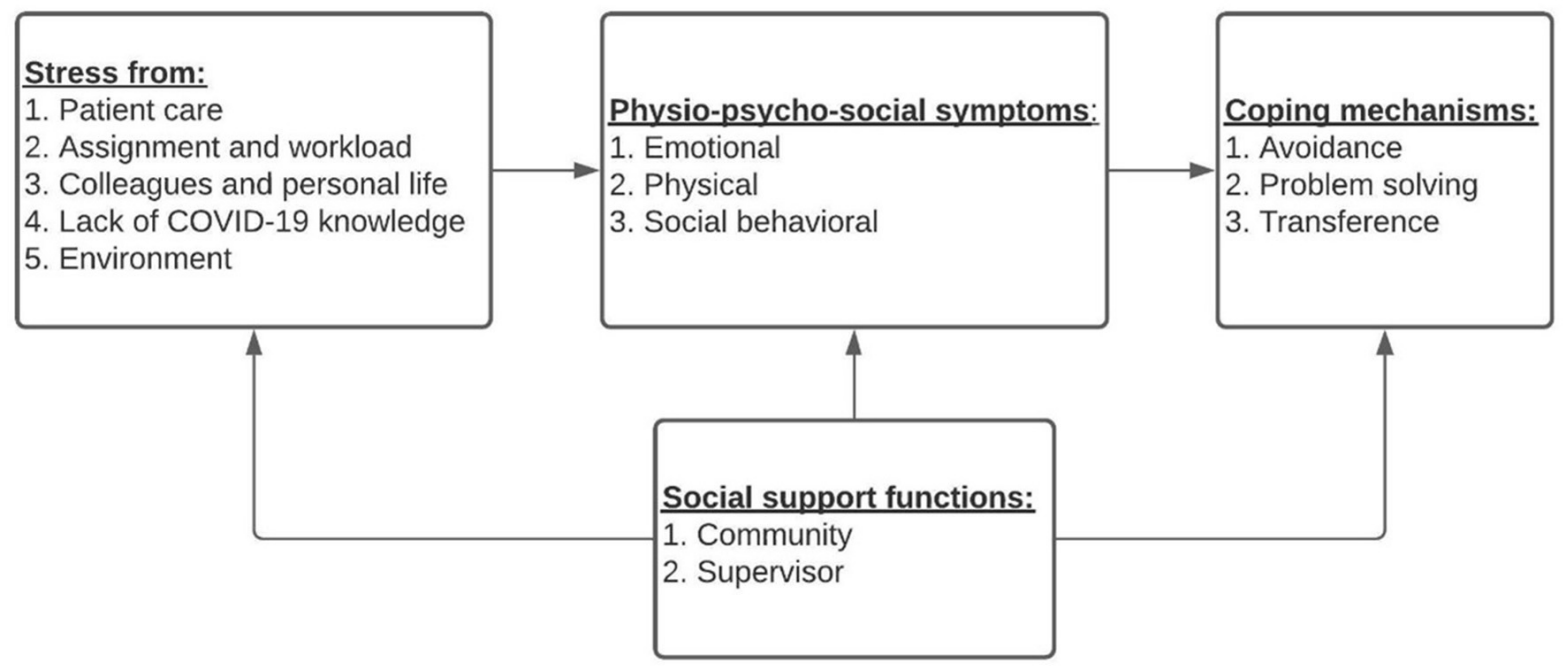
Stress-symptom-support-coping framework for frontline nurses during the COVID-19 pandemic.

Figure 1 illustrates this study's proposed framework, which examines a diverse array of coping mechanisms and the resulting approach nurses take to address sources of stress and physio-psychosocial symptoms. Coping mechanisms are not considered positive or negative, but rather “effective” or “ineffective” at reducing the psychosocial symptoms and stressors experienced by the nurse (32). Scholars familiar with stress and coping studies will note the similarities to studies by researchers such as Folkman et al. (33), specifically the concept that organizational support can theoretically influence other domains simultaneously (24, 34, 35). These models suggest that the psychosocial symptoms and stressors experienced by nurses, mediated by organizational support, influence how nurses cope with their stress. As coping mechanisms are effectively a nurse's sense-making process when working in a stressful environment, this model relates stress appraisal as an ongoing process that connects stressors and symptoms to appropriate coping mechanisms. Because nurses can anticipate the impact of stressful experiences based on previous experiences, the model considers how nurses pursue coping strategies before experiencing physio-psychosocial symptoms.

This paper seeks to bridge our understanding of stress, support, and coping mechanisms by examining frontline nurses in Alabama (2, 10, 20, 33). It aims to explore nurses' mental health in Alabama hospitals during the COVID-19 outbreak and the impact of organizational and social support on nurse stress levels, physio-psychosocial responses, and coping strategies employed.

## Methods

A cross-sectional survey was developed and distributed to nurses working with COVID-19-infected patients in hospital settings in Alabama. A total of 232 frontline nurses responded over the period of May 6, 2020–June 30, 2020. Nurses were invited to participate through information posted about the study on social media platforms, such as LinkedIn, and by encouraging nurses to share information about the study. An online link to the survey was shared with nurses who showed interest. The study's inclusion criteria specified only Alabama nurses working directly with COVID-19 patients in the 3 months prior to the data collection start date.

### Ethical Considerations

This study was approved by the Ethics Committee of XX University in accordance with the Declaration of Helsinki (IRB protocol reference: 20–238 EX 2005). Participants were notified about the aims of the project and the risks that might be associated with the survey on the first page, and a consent form was provided. Participants were notified that no identifiable information would be collected, and they agreed to participate in the study by completing the survey, and participants were compensated for their time if they choose to continue and take the survey ($20).

### Questionnaire Development

Principal items were developed based on a questionnaire designed by Lee et al. (36) to investigate medical staff during the 2003 SARS epidemic. Further, pandemic-specific questions were taken from an instrument by Cai et al. (37) that was used to examine frontline nurses in China. Finally, the questionnaire is a continuation of instruments created by Ali et al. (2) and Cole et al. (20), who investigated major psychological stressors and organizational resources that impact the stress and turnover intentions of frontline nurses in Alabama.

A total of 79 items were developed for the four domains: stressors, physio-psychosocial symptoms, coping, and support, see Figure 1. This list of questions was distributed to a group of experts in the field with research experience (four ICU nurses, two general nurses, two nursing faculty, one public health expert, and two nurse managers). After 1 week, a virtual focus session was conducted with a group of experts to discuss the preliminary list of items. In response to the experts' feedback, the social support items were revised for clarity.

The questionnaire instrument included *demographic and work-related questions* (9 items). *Stress from working as a frontline nurse* was captured using 29 items divided into five constructs: stress from taking care of patients, stress from assignments and workload, stress from colleagues and personal life, stress from a lack of knowledge about COVID-19, and stress from the environment. *Frontline nurse perceptions of physio-psychosocial symptoms* were captured using 15 items divided into three constructs: emotional symptoms, physical symptoms, and social behavioral symptoms. *Nurse perceptions of social support* was captured using 14 items divided into two constructs: community/social support and supervisor support. Finally, *coping strategies employed by nurses* were captured using 12 items divided into three constructs: avoidance, problem solving, and transference. The perception items were measured on a five-point Likert scale (from strongly disagree to strongly agree). A questionnaire template is provided in this study.

The survey instrument was pilot tested with 15 nurses working in local nurses who were invited through personal connections. Reliability was assessed using Cronbach's alpha, which reflects the interrelatedness among the items in each construct. A Cronbach's alpha of the constructs' values was within the acceptable range (α > 0.70; see **Table 2**).

### Operationalization of Domain Constructs

#### Stressors

##### Stress From Taking Care of Patients

Stress related to taking care of COVID-19 patients is well documented (2, 20, 24, 37). These questions are designed to capture aspects of stress resulting from working directly with patients infected with COVID-19.

##### Stress From Assignments and Workload

The COVID-19 pandemic requires nurses to provide various levels of healthcare to highly infectious patients. These assignments and tasks may not directly involve COVID-19, but are certainly impeded by the social distancing requirements and patient acuity resulting from the patient's infection (2, 20, 24, 37). This construct is used to capture the stress resulting specifically from patient assignment and the resulting workload.

##### Stress From Colleagues and Personal Life

Frontline nurses are not only afraid of working with COVID-19 patients, they are also afraid of getting their colleagues infected (2, 20, 24, 37). These questions are used to assess stress related to the fear of COVID-19 infections that negatively impact their colleagues and personal lives.

##### Stress From a Lack of Knowledge About COVID-19

Specific factors can exacerbate the difficulty of providing treatment (2, 20). However, due to the lack of information during the initial months of the pandemic, nurses experienced periods of time when information was scarce, healthcare standards were rapidly changing, and media coverage provided pessimistic outlooks on health capacity (2, 20). Questions related to this construct were designed to capture the uncertainty nurses felt in relation to stress from a lack of knowledge during the pandemic.

##### Stress From the Environment

Constant media coverage of certain topics, such as PPE shortages and ventilator shortages, may cause nurses to feel more anxious about their next or current shift (2, 20, 24). Items related to this construct were designed to capture the stress caused by nurses who perceived gaps in their work environments.

#### Physio-Psychosocial Symptoms

##### Emotional Symptoms

The fear, anxiety, and stress reported by nurses during the pandemic have been well documented (2, 20). This construct was designed to capture the mental and emotional experiences of working as a frontline nurse during the pandemic.

##### Physical Symptoms

Studies related to nurse occupational stress suggest that high levels of stress can cause adverse physical symptoms (2, 20). Intense anxiety, insomnia, poor diet, and headaches can all be triggered or exacerbated by stressful experiences related to working during the pandemic (2, 20, 24, 37). This construct was designed to capture the adverse physical symptoms reported by frontline nurses.

##### Social Behavioral Symptoms

Due to the social distancing requirements of the COVID-19 pandemic, there is both public and professional pressure to avoid becoming infected. This causes nurses to fear becoming infected, as they might pass on the virus by working as an asymptomatic carrier (2, 20, 24, 37). This construct was designed to capture the perception of adverse social conditions while working as a frontline nurse.

#### Support

##### Supervisor Support

Supervisor support is defined as the informal support and professional guidance frontline nurses receive from their supervisors to cope with stressful situations (38). This construct was designed to capture the extent to which supervisor support contributes to the reduction of stress and physio-psychosocial symptoms.

##### Community Support

Community support is defined as the organized or informal support received by frontline nurses from family members, friends, neighbors, religious organizations, community programs, cultural and ethnic organizations, and other support groups or organizations outside their workplace (24). This construct is used to measure the extent to which frontline nurses rely on social support from outside the hospital to mitigate physio-psychosocial symptoms.

#### Coping

##### Avoidance

Avoidance refers to a coping strategy used by frontline nurses to distance themselves from the source of their stress (2, 6, 24, 31, 33, 35, 39). Avoidance is used to measure the extent to which frontline nurses attempt to avoid rather than engage with their sources of stress.

##### Problem Solving

Problem solving refers to the coping strategy that involves frontline nurses engaging in a series of deductive steps to understand how to address and mitigate the source of their stress (2, 6, 24, 31, 33, 35, 39). Problem solving is used to measure the extent to which frontline nurses attempt to “figure out” and address their stress as a coping strategy.

##### Transference

Transference refers to a coping strategy that involves frontline nurses engaging in interpersonal communication with a professional therapist (2, 6, 24, 31, 33, 35, 39). Transference is used to measure the extent to which frontline nurses attempt to seek psychological therapy to address their stress as a coping strategy.

## Results

The statistical analysis included descriptive statistics of the demographic factors examined in the survey. Next, a two-way ANOVA, Pearson's correlations of the constructs, and regression analysis were used to analyze the domains and constructs. Finally, the direct and indirect effects of the domains tested were analyzed to determine the influences of social support and coping mechanisms on occupational stress and associated physio-psychosocial symptoms.

### Descriptive Statistics of Demographic Variables

Table 1 provides the descriptive statistics of the demographic variables. The results show that respondents were relatively young, with 43.5% (*n* = 101) under 30 years old. They suggest that slightly over half 50.9% (*n* =118) of nurses were less experienced while 10.8% (*n* = 25) were senior. Further, there is a roughly even proportion of married nurses at 47.0% (*n* = 109) compared to the 44.8% (*n* = 104) who have never married. It should be noted that 68.1% (*n* = 158) of our respondents had at least one child, while roughly 31.9% (*n* = 78) had no child. Overall, most nurse respondents were female 90.9% (*n* = 211). Lastly, a third of the nurse respondents specialized as general nurses (30.6%; *n* = 71), making up the largest single specialization in the sample.

**Table 1:** Descriptive statistics.

	**Respondents**	**Percent (*N* = 232)**
**Age**
1 = <30	101	43.5
2 = > 30	48	20.7
3 = > 40	29	12.5
4 = > 50	54	23.3
SD	1.21	
**Gender**
1 = M	21	9.1
2 = F	211	90.9
SD	0.288	
**Ethnicity**
1= White	211	90.9
2 = African American (non-Hispanic)	21	9.1
SD	0.288	
**Marital status**
1 = Married	109	47
2 = Divorced	19	8.2
3 = Single (never married)	104	44.8
SD	1.92	
**Have children?**
1 = No	158	68.1
2 = Yes	78	31.9
SD	0.467	
**Seniority**
1 = <10 years of experience	118	50.9
2 = > 10 years of experience	89	38.4
3 = > 15 years of experience	25	10.8
SD	0.677	
**Specialty**
1 = General nurse	71	30.6
2 = ICU	64	27.6
3 = OR	35	15.1
4 = ER	30	12.9
5 = Other	32	13.8
SD	1.399	
**Shift**
1 = Morning	158	68.1
2 = Evening	74	31.9
SD	0.467	

### ANOVA: Analysis of Demographic Variables and Constructs

Appendix 1 (see Section 1) provides the results of the two-way ANOVA between the demographic variables and the domain constructs.

#### Stressors

##### Stress From Taking Care of Patients

The analysis of variance between the demographic variables and stress from taking care of patients showed that gender (*p* < 0.01), having children (*p* < 0.01), and specialty (*p* < 0.01) were all statistically significant predictors of patient care-related stress for frontline nurses. Nurses between the ages 41–50 (*p* < 0.01) and nurses with over 10 years of experience showed significantly lower stress levels (*p* < 0.05). In general, more than 65% of the nursing staff reported high stress levels due to taking care of patients infected with COVID-19.

##### Stress From Assignments and Workload

Gender (*p* < 0.01), marital status (*p* < 0.01), and specialty (*p* < 0.05) all demonstrated significant relationships with stress from assignments and workload. Nurses aged 31–40 (*p* < 0.01), nurses aged 30 and younger (*p* < 0.01) and nurses who had no children reported significantly higher stress levels (*p* < 0.05). Nurses with more than 10 years of experience showed significantly lower mean stress levels (*p* < 0.05), and more than 80% of respondents reported high stress levels due to assignments or workload in general.

##### Stress From Colleagues, Staff, and Personal Life

It was found that marital status (*p* < 0.01), having children (*p* < 0.01), and specialty (*p* < 0.05) were all significantly related to stress from colleagues, staff, and personal life. Female respondents showed significantly higher mean stress levels (*p* < 0.01). Nurses aged 50 and older (*p* < 0.01) and nurses with over 10 years of experience reported lower stress levels (*p* < 0.01). Overall, around 70% of respondents reported higher stress (>3) resulting from worry or concern about colleagues or family members.

##### Stress From a Lack of Knowledge About COVID-19

It was found that gender (*p* <0.01), seniority (*p* < 0.01), and specialty (*p* < 0.01) were all significantly related to stress from a lack of knowledge about COVID-19. Nurses that were never married reported significantly higher mean stress levels (*p* < 0.01). Nurses aged 41–50 reported significantly lower mean stress levels (*p* < 0.01). Overall, around 70% of nurses in the study reported stress levels higher than 3 on the Likert scale.

##### Stress From the Environment

It was found that seniority (*p* < 0.01), and specialty (*p* < 0.01) were statistically significant predictors of stress from the environment. Female nurses showed significantly higher stress levels than their male counterparts (*p* < 0.01). Nurses aged 50 and older (*p* < 0.01), as well as married nurses (*p* < 0.01), reported significantly lower stress levels. Around 77% of nurses in the study reported a high level of stress resulting from their environment.

#### Physio-Psychosocial Symptoms

##### Emotional Symptoms

The analysis of variance between the demographic variables and emotional symptoms showed that gender (*p* < 0.01), marital status (*p* < 0.01), seniority (*p* < 0.01), specialty (*p* < 0.01), and shift (*p* < 0.05) were all s tatistically significant characteristics of frontline nurses. Nurses aged 50 and older reported significantly lower emotional symptoms (*p* < 0.01).

##### Physical Symptoms

It was found that age (*p* < 0.01), seniority (*p* < 0.01), specialty (*p* < 0.01), and shift (*p* < 0.05) were all related to physical symptoms. Female nurses (3.33, *p* < 0.01) and nurses who were never married (3.46, *p* < 0.01) reported significantly higher mean physical symptom levels.

##### Social Behavioral Symptoms

It was found that marital status (*p* < 0.01), seniority (*p* < 0.01), and specialty (*p* < 0.01) were all related to frontline nurse social behavioral symptoms. Nurses aged 50 and older reported significantly lower mean social behavioral symptoms (*p* < 0.05). Nurses aged 31–40 years (*p* < 0.05) and female nurses reported significantly higher mean social behavioral symptom levels (*p* < 0.01).

#### Coping

##### Avoidance

The analysis of variance between the demographic variables and avoidance as a coping strategy showed that gender (*p* < 0.01) and specialty (*p* < 0.01) were significant predictors. Nurses aged 50 and older reported significantly lower mean avoidance usage (0.35, *p* < 0.01).

##### Problem Solving

It was found that specialty (*p* < 0.01) was significantly related to frontline nurses' tendency to use problem solving as a coping strategy. Nurses aged 31–40 (*p* < 0.01), respondents who had never been married (*p* < 0.01), and female respondents (*p* < 0.01) showed significantly higher mean problem-solving usage.

##### Transference

It was found that age (*p* < 0.05), marital status (*p* < 0.05), specialty (*p* < 0.05) having children (*p* < 0.05), and shift (*p* < 05) were all statistically significant predictors of frontline nurses' tendency to use transference as a coping strategy. Nurses with over 10 years of experience reported significantly lower mean transference usage (*p* < 0.01).

#### Support

##### Supervisor Support

The analysis of variance between the demographic variables and supervisor support found that specialty (*p* < 0.05) was a statistically significant predictor of frontline nurses' supervisor support. Nurses aged 30 and younger (*p* < 0.01) and nurses with morning shifts reported significantly higher mean levels of supervisor support (*p* < 0.05). Respondents that had been divorced showed significantly lower mean levels of supervisor support (*p* < 0.05). Overall, only 37% of the respondents reported receiving support from their supervisors.

##### Community Support

It was found that specialty (*p* < 0.05) and shift (*p* < 0.05) were statistically significant predictors of reliance on community support. Nurses aged 50 and older (*p* < 0.01), and nurses who had been divorced showed higher levels of community support (*p* < 0.05). Nurse respondents with more than 10 years of experience reported significantly lower mean levels of community support (*p* < 0.01). In general, around 44% of nurses reported a high level of community support.

### ANOVA: Analysis of Demographic Variables and Domains

Appendix 1 (see Section 2) provides the results of the two-way ANOVA between the demographic variables and domain scores.

#### Stressors Domain

The analysis of variance between the demographic variables and the stressors domain showed that gender (*p* < 0.01), marital status (*p* < 0.01), and specialty (*p* < 0.01), were all statistically significant predictors of stress for frontline nurses. Nurses aged 41–50 (*p* < 0.01), nurses aged 50 and older (*p* < 0.01), and nurses with more than 10 years of experience reported significantly lower levels of stress (*p* < 0.01).

#### Physio-Psychosocial Symptoms Domain

It was found that specialty (*p* < 0.01), and shift (*p* < 0.05), were all statistically significant predictors of frontline nurse physio-psychosocial symptoms. Nurses aged 50 and older (*p* < 0.01), nurses who had been divorced (*p* < 0.01), and nurses with over 10 years of experience (*p* < 0.01) reported significantly lower mean levels of physio-psychosocial symptoms.

#### Coping Domain

It was found that specialty (*p* < 0.01), and shift (*p* < 0 .05), were statistically significant predictors of frontline nurse coping habits. Nurses aged 31–40 (*p* < 0.01) and female respondents (*p* < 0.01) reported significantly higher mean use of coping strategies. Nurses that were divorced (*p* < 0.01) and nurses with over 10 years of experience reported significantly lower mean coping strategy usage (*p* < 0.01).

#### Support Domain

It was found that nurses aged 31–40 (p < 0.01) and nurses with over 10 years of experience showed significantly lower mean levels of support (p < 0.05). Nurses who had never married reported significantly higher mean levels of support (p < 0.01).

### Correlation Analysis

Pearson correlations were checked (Table 2) to investigate the correlations between this study's domain constructs. With the largest correlation coefficient, stress from nurses' personal lives correlated significantly and positively with stress from task workload (r _(4)_ = 0.715, p < 0.001). Stress from a lack of knowledge correlated significantly and positively with stress from workload (r _(4)_ = 0.692, p < 0.001). Stress from the environment correlated strongly and positively with stress from personal life (r _(4)_ = 0.686, p < 0.001), stress from patients correlated significantly and positively with stress from workload (r _(4)_ = 0.656, p < 0.001), and stress from lack of knowledge correlated significantly and positively with stress from workload (r _(4)_ = 0.655, p < 0.001).

**Table 2:** Pearson correlations between domain constructs.

		**M**	**SD**	**∞**	**1**	**2**	**3**	**4**	**5**	**6**	**7**	**8**	**9**	**10**	**11**	**12**	**13**
1	Stress from patients	3.27	0.92	0.732	1												
2	Stress from workload	3.63	0.66	0.81	0.656**	1											
3	Stress from personal life	3.53	0.89	0.769	0.644**	0.715**	1										
4	Stress from lack of knowledge	3.53	0.82	0.856	0.692**	0.655**	0.575**	1									
5	Stress from environment	3.62	0.83	0.782	0.590**	0.596**	0.686**	0.653**	1								
6	Emotional symptoms	2.7	0.95	0.887	0.515**	0.514**	0.522**	0.613**	0.573**	1							
7	Physical symptoms	3.31	0.76	0.708	0.502**	0.376**	0.552**	0.465**	0.560**	0.655**	1						
8	Social behavioral symptoms	2.41	0.96	0.774	0.427**	0.395**	0.472**	0.336**	0.544**	0.508**	0.507**	1					
9	Supervisor support	2.71	0.75	0.76	0.05	0.121	0.064	0.035	0.082	−0.065	−0.117	−0.092	1				
10	community support	2.8	0.64	0.794	−0.008	0.029	−0.197**	0.006	−0.067	−0.11	−0.166*	−0.152*	−0.017	1			
11	Coping strategy avoidance	1.03	0.94	0.78	0.258**	0.348**	0.428**	0.295**	0.397**	0.248**	0.119	0.262**	−0.029	−0.059	1		
12	Coping strategy problem solving	2.67	1.34	0.699	0.433**	0.333**	0.428**	0.606**	0.606**	0.450**	0.424**	0.424**	0.028	−0.130*	0.389**	1	
13	Coping strategy transference	1.84	0.74	0.72	0.009	0.085	0.078	0.007	−0.165*	0.133*	0.161*	0.138*	−0.042	−0.109	0.013	0.016	1

*M, Mean; SD, Standard deviation; α, Cronbach's Alpha reliability*.

*p < 0.05 and **p < 0.01.

### Regression Analysis of Demographic Variables and Domains

Appendix 1 (see Section 3) provides the regression analyses of demographic variables and domain scores.

#### Stressors Domain

Age was found to be statistically significant in two ways: stress was highest among nurses aged 31–40 years old (p < 0.01) and lowest among nurses aged 41–50 years old (p < 0.05). Being female corresponded to a significant increase in stress, by almost a whole unit (p < 0.01). Respondents who reported being divorced had significantly lower stress (p < 0.01), while never having married corresponded with high stress (p < 0.01). Nurses with more than 10 years of work experience reported higher stress (p < 0.01), and lower stress levels were reported by nurses who worked in the operating room (p < 0.05), emergency room (p < 0.05), and other (p < 0.01).

#### Physio-Psychosocial Symptoms Domain

Physical and psychosocial symptoms were higher among nurses aged 31–40 years old (p < 0.01) and lower among nurses aged 41–50 years old (p < 0.05). Being female corresponded to significantly higher (more than one unit) symptoms (p < 0.01). Divorced nurses reported less symptoms (p < 0.01). Nurses who had more than 10 years of work experience reported more symptoms (p < 0.01), while those with <15 years of experience had lower symptoms (p < 0.01). Nurses working in operating rooms (p < 0.01), and “other” specialties (p < 0.01) had significantly lower symptoms, and working the night shift was related to higher physio-psychosocial symptoms (p < 0.05).

#### Coping Domain

Around 67% of nurses reported using at least one avoidance coping mechanism, 84% reported using problem-solving coping techniques, and 95% reported a form of transference coping mechanism. However, no one of the respondents reported seeking help from a psychologist as a coping mechanism. Nurses working in operating rooms (p < 0.05), emergency rooms (p < 0.05), and other (p < 0.01) reported lower use of coping strategies, as did nurses working on the night shift (p < 0.05). Being female corresponded to a significantly higher (more than one unit) usage of coping strategies (p < 0.01), as did being African American (p < 0.01). Being divorced corresponded with significantly lower use of coping strategies (p < 0.01). Overall, single (never married) nurses reported a significant and higher use of coping mechanisms (p < 0.01), and more problem solving coping.

#### Social Support Domain

The perceived importance of social support was lower among nurses aged 31–40 (p < 0.01) and higher among nurses aged 41–50 (p < 0.05). Nurses with more than 15 years of work had significantly and married nurses reported significantly higher social support (p < 0.05).

### Mediational Analyses

**Figure 2:**
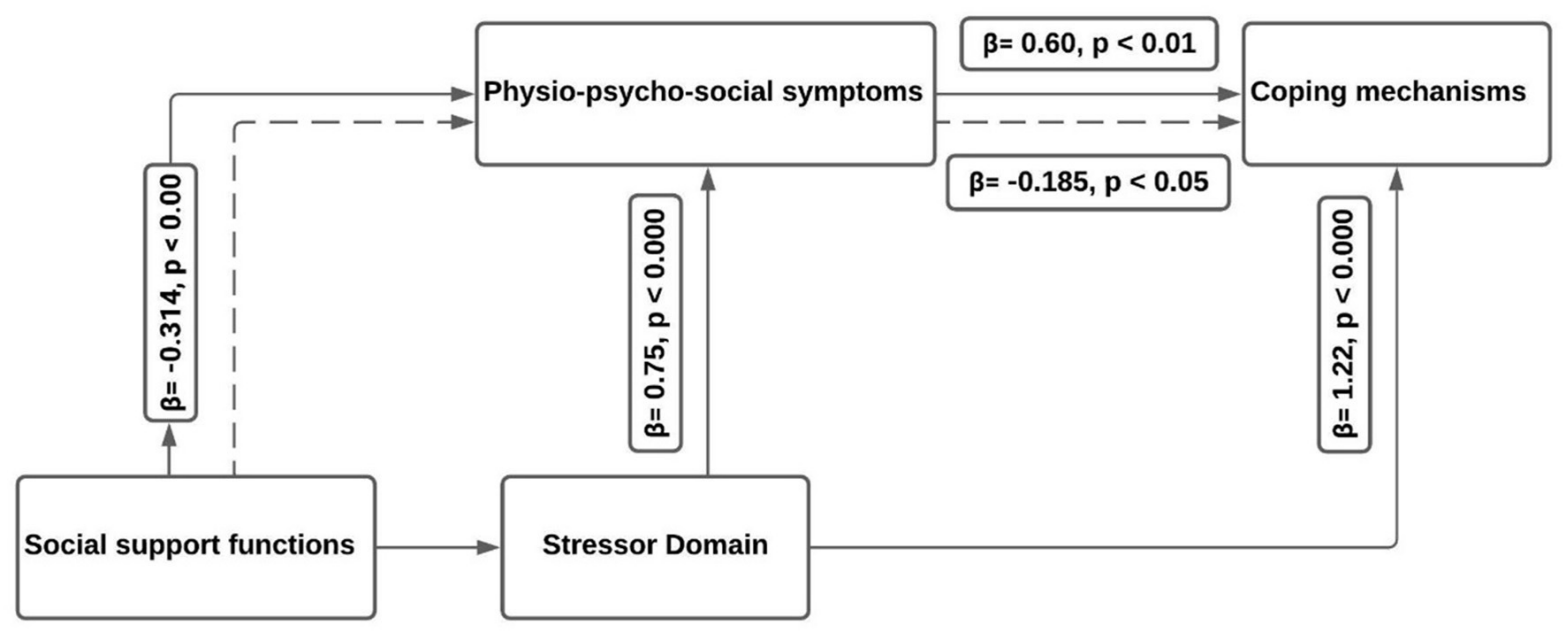
Revised framework for frontline nurse stress, symptom, support, and coping.

Table 3 reports the results for the direct and indirect effects of support on nurses' coping strategies. Figure 2 provides an illustration of the framework in light of the empirical results.

**Table 3:** Bootstrapping results for the direct and indirect effect of domains.

**Direct effect**	**coeff**	**se**	**t**	**p-value**	**LLCI**	**ULCI**
Support → Stressor	0.0298	0.0935	0.3183	0.7505	−0.1544	0.2140
Support → Symptoms*	−0.3140	0.0691	−4.5457	< 0.001	−0.4501	−0.1779
Stressor → Symptoms*	0.7480	0.0487	15.3569	< 0.001	0.6520	0.8439
Support → Coping	−0.3104	0.2344	−1.3242	0.1868	−0.7724	0.1515
Symptoms → Coping*	0.5918	0.2148	2.7550	0.0063	0.1685	1.0150
Stressor → Coping*	1.2207	0.2256	5.4117	< 0.001	0.7762	1.6651
**Level of confidence for all confidence intervals in output: 95%**.				
**Indirect effect**	**Effect**	**BootSE**	**BootLLCI**	**BootULCI**
Support → Stressor → Coping	0.0363	0.1186	−0.1917	0.2766
Support → Symptoms → Coping*	−0.1858	0.0693	−0.3302	−0.0601
Support → Stressor → Symptoms → Coping	0.0132	0.0451	−0.0827	0.1032

N = 232; LL, Lowe limit; UL, Upper limit; *p < 0.05.

#### Direct Effect

This analysis shows the direct relationship between independent and dependent variables. Table 3 shows that there is no direct effect between the independent variable (support) and dependent variable (coping strategies) (*P* = 0.1868). In other words, the “c” coefficient is not statistically significant. It also reports that the direct effect between support and symptoms is β = −0.3140 (*P* < 0.001). In other words, “m1” coefficient is statistically significant. It demonstrates that direct effect between mediators (stressor and symptoms) is β = 0.7480 (*P* < 0.001). The direct effect between mediators (Symptoms and stressor) and coping strategies are β = 0.5918 (*p* = 0.1685) and β = 1.2207 (*P* < 0.001), respectively. So, “b1” and “b2” coefficients are statistically significant.

#### Indirect Effect

This part of the results tests the indirect relationship between independent and dependent variables. The symptom is the only mediator who mediates the relationship between support and coping strategies. The indirect effect is equal to−0.1858 with a 95% bootstrap confidence interval; that is, the indirect effect is statistically significant at alpha 0.05 (*P* < 0.05).

## Discussion

This study aimed to investigate the relationships between frontline nurse stress, physio-psychosocial symptoms, and coping behaviors employed by nursing staff during the COVID-19 pandemic. Additionally, this study aimed to examine the relationships between coping strategies and social support on adverse symptoms and their related stressors. The study's results ultimately reflect the age and occupational differences in how frontline nurses perceive and engage in support and coping to reduce physio-psychosocial symptoms. Furthermore, the results reflect how certain stressors and symptoms are associated with specific coping behaviors. Finally, the results suggest a causal relationship in which nurses appraise their stress prior to symptoms and choose their coping strategies based on the symptoms felt.

### Age and Specialty: Frontline Nurse Stress, Coping, and Support

This study found that factors related to aging significantly contributed to stress from personal life, emotional symptoms, physical symptoms, and social symptoms. For example, being over 50 years old was associated with a significant positive relationship with social support. It seems clear from this data that age differences at least partially determined frontline nurses' preferences in how they coped with occupational stress factors. This finding is consistent with Ali et al. (2). This is perhaps why older nurses preferred social relationships and transference over more individual forms of coping, such as problem-solving (6, 33, 35, 40). Overall, these results indicate that age differences influence how nurses perceive the efficacy of coping strategies and social support.

According to the results of this study, the specialty was found to be a significant demographic factor affecting stress, symptoms, and coping strategy usage. The results show that nurses in specialties such as OR, ER, and pediatrics reported having significantly lower stress, physio-psychosocial symptoms, and use of coping strategies than general nurses. Interestingly, specialties such as ICU and OR reported requiring substantially more support than general nurses. These findings suggest that specialties requiring less patient exposure result in greater stress levels, which is consistent with Ali et al. (2) and Cai et al. (37).

Specialties involving more technical procedures, such as OR, and those involving more uncertain and volatile patients, such as ICU, seem to require more social support (2, 24).

### Gender: Frontline Nurse Stress, Coping, and Support

The results indicated that female nurses have significantly higher stress and Physio-psycho-social emotional symptoms than male nurses. Female nurses reported a higher level of anxiety, sadness, and depression, but a higher score for problem-solving techniques was reported by female nurses. In general, female nurses indicated higher use of coping strategies than their male counterparts. This is consistent with Ali et al. (2) and Huang et al. (41), who reported that female nurses are more likely to suffer from psychological problems and report a higher level of stress (42, 43). This might be because female nurses spend more time and effort communicating and providing mental support to patients and their life and family responsibilities (41, 42). In addition, female nurses reported a higher community and organizational support level. This observation might be because females generally have more social responsibilities and engage in social and family activities more than males. Also, related to Hamdan-Mansour et al. (44), female nurses report significantly higher stress. Therefore, they may be more in need of community and organizational support.

### Seniority: Frontline Nurse Stress, Coping, and Support

Although stress seems problematic for all nurses' specialties, little is known about nursing seniority differences. López-López et al., (45) reported that professional seniority variables contribute to burnout and stress development in nurses. More experienced nurses have reported less stress levels, which may be related to having more years of training and dealing with patients' related stress. In addition, these nurses reported significantly lower mean levels of physio-psychosocial symptoms and needed support.

This study shows that nurses with over ten years of experience reported significantly lower mean coping strategy usage. With experience, nurses may have developed greater emotional and mental resilience out of job necessity (2). Younger nurses reported a higher level of organizational and social support. They are mostly less experienced and request more support from supervisors due to a lack of confidence than their counterparts. In addition, Kath et al., (46) declared age had a significant positive relationship with autonomy. Another reason could be that the younger nurses work with an older ones (46). These could be the reasons for reporting more support levels among younger nurses. In contrast, Laal and Aliramaie, (47) reported that junior and senior staff had no difference in applying positive or negative responses to cope with stress.

### Marital Status: Frontline Nurse Stress, Coping, and Support

In general, all nurses reported using a sort of problem-solving coping strategy. More than 62% of younger nurses reported thoughts of leaving their job. In contrast, single nurses reported using avoidance coping strategies more than married ones. In conclusion, married nurses were indicated to have lower stress levels during the COVID-19 pandemic. This is consistent with Ali et al. (2) findings. Unsurprisingly, transference coping strategies were considered more by married nurses. This could be related to the support they receive from their partners and the ability to transfer and redirect the stress to their partner.

These results help to understand better why nurses feel the need for additional social and organizational support in light of greater uncertainty and why some specialties require more support than others in pandemic circumstances. However, further research into the differences among nurses' perceptions of support, in general, is needed to highlight potential gaps in how current and future nurses perceive the efficacy of social support.

### Stressors, Physio-Psychosocial Symptoms, Support, and Coping

Figure 2 provides an updated framework that considers the insights from the bootstrapping results. As expected, stressors have a significant direct effect on harmful Physio-psychosocial symptoms. Further, harmful symptoms have a significant positive impact on the need for coping strategies. The direct effects provide a practical illustration of the ways stress leads to symptoms and how symptoms lead to coping strategies. Additionally, it suggests that nurses can appraise their stress more quickly than scholars have theorized, as stress and symptoms (24, 33). Finally, greater social support may reduce nurses' reliance on coping strategies.

## Conclusion

This study found that both social support and physio-psychosocial symptoms contributed to the use of coping strategies. This study reveals that nurses who experience a higher level of stress are more likely to experience poor physio-psycho-social symptoms and negatively cope with the stress. This study demonstrates that organizational and social support could reduce stress intensity and improve the physio-psycho-social status by reducing the harmful symptoms.

This study provides further evidence of the importance of organizational support in helping alleviate the harmful physio-psychosocial symptoms experienced by nurses. The study helped identify unique patterns related to nursing support during the pandemic. However, this study lacked sample diversity, such as gender. Gender-related findings could not be generalized because of the relatively small sample size of male participants in this study.

## Data Availability Statement

The raw data that support the findings of this study are available from the corresponding author upon reasonable request.

## Ethics Statement

The studies involving human participants were reviewed and approved by Auburn University IRB Review Committee (IRB protocol reference: 20–238 EX 2005). The patients/participants provided their written informed consent to participate in this study.

## Author Contributions

HA and DA designed the study and collected the data. HA, YF, and SH analyzed the collected data and MH helped with the data analysis. All authors drafted the manuscript, made necessary changes and revisions to the manuscript, and approved the final version.

## Funding

The study was funded through HA start-up funds.

## Conflict of Interest

The authors declare that the research was conducted in the absence of any commercial or financial relationships that could be construed as a potential conflict of interest. The reviewer OA declared a shared affiliation with the author MH to the handling editor at time of review.

## Publisher's Note

All claims expressed in this article are solely those of the authors and do not necessarily represent those of their affiliated organizations, or those of the publisher, the editors and the reviewers. Any product that may be evaluated in this article, or claim that may be made by its manufacturer, is not guaranteed or endorsed by the publisher.
