# Supplementary material for: Investigating Frontline Nurse Stress: Perceptions of Job Demands, Organizational Support, and Social Support During the Current COVID-19 Pandemic
Source: Front Public Health. 2022 May 25;10:839600. doi: 10.3389/fpubh.2022.839600 (PMC9204268; doi:10.3389/fpubh.2022.839600)
Supplement: Supplementary file 1 [file Data_Sheet_1.docx]

**Appendix 1- Section 1**

**Stressors**

| 1: Stress from taking care of patients |  |  |  |  |  |
| --- | --- | --- | --- | --- | --- |
| Analysis of Variance |  |  |  |  |  |
| Source | Adjusted Sum of Square | DF | Adjusted Mean Square | F-Value | Sig |
| Age: | 9.126 | 3 | 3.0418 | 6.19 | 0.000 |
| Gender | 8.495 | 1 | 8.4951 | 17.3 | 0.000 |
| Ethnicity | 1.25 | 1 | 1.2496 | 2.54 | 0.112 |
| Marital status | 0.971 | 2 | 0.4856 | 0.99 | 0.374 |
| Having children | 2.309 | 1 | 2.309 | 4.7 | 0.031 |
| Seniority | 6.391 | 2 | 3.1953 | 6.51 | 0.002 |
| Specialty | 20.056 | 4 | 5.0139 | 10.21 | 0.000 |
| Shift | 1.733 | 1 | 1.7334 | 3.53 | 0.062 |

| 2: Stress from assignments and workload |  |  |  |  |  |
| --- | --- | --- | --- | --- | --- |
| Analysis of Variance |  |  |  |  |  |
| Source | Adjusted Sum of Square | DF | Adjusted Mean Square | F-Value | Sig |
| Age: | 7.026 | 3 | 2.34199 | 11.67 | 0.000 |
| Gender | 5.369 | 1 | 5.36901 | 26.76 | 0.000 |
| Ethnicity | 0.391 | 1 | 0.39108 | 1.95 | 0.164 |
| Marital status | 14.251 | 2 | 7.12527 | 35.51 | 0.000 |
| Having children | 2.056 | 1 | 2.0564 | 10.25 | 0.002 |
| Seniority | 17.019 | 2 | 8.50934 | 42.41 | 0.000 |
| Specialty | 4.03 | 4 | 1.00753 | 5.02 | 0.001 |
| Shift | 0.005 | 1 | 0.00538 | 0.03 | 0.87 |

| 3: Stress from colleagues, staff, and personal life |  |  |  |  |  |
| --- | --- | --- | --- | --- | --- |
| Analysis of Variance |  |  |  |  |  |
| Source | Adjusted Sum of Square | DF | Adjusted Mean Square | F-Value | Sig |
| Age: | 19.93 | 3 | 6.6433 | 18.98 | 0.000 |
| Gender | 8.681 | 1 | 8.6814 | 24.81 | 0.000 |
| Ethnicity | 0.914 | 1 | 0.9139 | 2.61 | 0.108 |
| Marital status | 14.051 | 2 | 7.0255 | 20.08 | 0.000 |
| Having children | 5.369 | 1 | 5.3687 | 15.34 | 0.000 |
| Seniority | 38.721 | 2 | 19.3606 | 55.33 | 0.000 |
| Specialty | 7.288 | 4 | 1.8221 | 5.21 | 0.001 |
| Shift | 1.077 | 1 | 1.077 | 3.08 | 0.081 |

| 4: Stress from a lack of knowledge about Covid-19 |  |  |  |  |  |
| --- | --- | --- | --- | --- | --- |
| Analysis of Variance |  |  |  |  |  |
| Source | Adjusted Sum of Square | DF | Adjusted Mean Square | F-Value | Sig |
| Age: | 7.363 | 3 | 2.4544 | 7.73 | 0.000 |
| Gender | 17.554 | 1 | 17.5539 | 55.32 | 0.000 |
| Ethnicity | 1.968 | 1 | 1.9684 | 6.2 | 0.014 |
| Marital status | 6.716 | 2 | 3.3579 | 10.58 | 0.000 |
| Having children | 0.314 | 1 | 0.3143 | 0.99 | 0.321 |
| Seniority | 6.074 | 2 | 3.037 | 9.57 | 0.000 |
| Specialty | 11.971 | 4 | 2.9928 | 9.43 | 0.000 |
| Shift | 0.219 | 1 | 0.2191 | 0.69 | 0.407 |

| 5: Stress from the environment |  |  |  |  |  |
| --- | --- | --- | --- | --- | --- |
| Analysis of Variance |  |  |  |  |  |
| Source | Adjusted Sum of Square | DF | Adjusted Mean Square | F-Value | Sig |
| Age: | 5.95 | 3 | 1.9832 | 5.96 | 0.001 |
| Gender | 17.804 | 1 | 17.8036 | 53.49 | 0.000 |
| Ethnicity | 0.207 | 1 | 0.207 | 0.62 | 0.431 |
| Marital status | 13.287 | 2 | 6.6436 | 19.96 | 0.000 |
| Having children | 1.02 | 1 | 1.0202 | 3.07 | 0.081 |
| Seniority | 16.497 | 2 | 8.2487 | 24.78 | 0.000 |
| Specialty | 13.526 | 4 | 3.3814 | 10.16 | 0.000 |
| Shift | 0.193 | 1 | 0.1931 | 0.58 | 0.447 |

**Physio-psycho-social symptoms**

| 1: Emotional symptoms |  |  |  |  |  |
| --- | --- | --- | --- | --- | --- |
| Analysis of Variance |  |  |  |  |  |
| Source | Adjusted Sum of Square | DF | Adjusted Mean Square | F-Value | Sig |
| Age: | 8.123 | 3 | 2.7077 | 4.78 | 0.003 |
| Gender | 17.879 | 1 | 17.8792 | 31.54 | 0.000 |
| Ethnicity | 2.8 | 1 | 2.8001 | 4.94 | 0.027 |
| Marital status | 9.587 | 2 | 4.7936 | 8.46 | 0.000 |
| Having children | 1.031 | 1 | 1.031 | 1.82 | 0.179 |
| Seniority | 13.642 | 2 | 6.8212 | 12.03 | 0.000 |
| Specialty | 17.726 | 4 | 4.4316 | 7.82 | 0.000 |
| Shift | 3.803 | 1 | 3.8034 | 6.71 | 0.01 |

| 2: Physical symptoms |  |  |  |  |  |
| --- | --- | --- | --- | --- | --- |
| Analysis of Variance |  |  |  |  |  |
| Source | Adjusted Sum of Square | DF | Adjusted Mean Square | F-Value | Sig |
| Age: | 20.209 | 3 | 6.7362 | 21.48 | 0.000 |
| Gender | 10.573 | 1 | 10.5735 | 33.72 | 0.000 |
| Ethnicity | 3.174 | 1 | 3.1738 | 10.12 | 0.002 |
| Marital status | 26.428 | 2 | 13.2139 | 42.14 | 0.000 |
| Having children | 1.118 | 1 | 1.1177 | 3.56 | 0.06 |
| Seniority | 23.127 | 2 | 11.5633 | 36.88 | 0.000 |
| Specialty | 5.959 | 4 | 1.4898 | 4.75 | 0.001 |
| Shift | 1.966 | 1 | 1.9659 | 6.27 | 0.013 |

| 3: Social behavioral symptoms |  |  |  |  |  |
| --- | --- | --- | --- | --- | --- |
| Analysis of Variance |  |  |  |  |  |
| Source | Adjusted Sum of Square | DF | Adjusted Mean Square | F-Value | Sig |
| Age: | 8.368 | 3 | 2.7894 | 4.78 | 0.003 |
| Gender | 20.761 | 1 | 20.7608 | 35.56 | 0.000 |
| Ethnicity | 0.216 | 1 | 0.216 | 0.37 | 0.544 |
| Marital status | 29.706 | 2 | 14.853 | 25.44 | 0.000 |
| Having children | 0.326 | 1 | 0.326 | 0.56 | 0.456 |
| Seniority | 6.986 | 2 | 3.4931 | 5.98 | 0.003 |
| Specialty | 18.108 | 4 | 4.5269 | 7.75 | 0.000 |
| Shift | 0.86 | 1 | 0.8598 | 1.47 | 0.226 |

**Coping Strategies**

| 1: D1-Avoidance |  |  |  |  |  |
| --- | --- | --- | --- | --- | --- |
| Analysis of Variance |  |  |  |  |  |
| Source | Adjusted Sum of Square | DF | Adjusted Mean Square | F-Value | Sig |
| Age: | 23.98 | 3 | 7.99339 | 13.69 | 0.000 |
| Gender | 6.836 | 1 | 6.83582 | 11.71 | 0.001 |
| Ethnicity | 9.027 | 1 | 9.02669 | 15.46 | 0.000 |
| Marital status | 0.824 | 2 | 0.41185 | 0.71 | 0.495 |
| Having children | 0.086 | 1 | 0.08587 | 0.15 | 0.702 |
| Seniority | 1.075 | 2 | 0.53768 | 0.92 | 0.4 |
| Specialty | 15.598 | 4 | 3.8995 | 6.68 | 0.000 |
| Shift | 0.552 | 1 | 0.55249 | 0.95 | 0.332 |

| 2: D2-Problem solving |  |  |  |  |  |
| --- | --- | --- | --- | --- | --- |
| Analysis of Variance |  |  |  |  |  |
| Source | Adjusted Sum of Square | DF | Adjusted Mean Square | F-Value | Sig |
| Age: | 28.961 | 3 | 9.6537 | 8.42 | 0.000 |
| Gender | 98.626 | 1 | 98.6264 | 85.98 | 0.000 |
| Ethnicity | 0.529 | 1 | 0.5291 | 0.46 | 0.498 |
| Marital status | 12.493 | 2 | 6.2467 | 5.45 | 0.005 |
| Having children | 1.198 | 1 | 1.198 | 1.04 | 0.308 |
| Seniority | 4.014 | 2 | 2.0068 | 1.75 | 0.176 |
| Specialty | 22.595 | 4 | 5.6487 | 4.92 | 0.001 |
| Shift | 1.665 | 1 | 1.6646 | 1.45 | 0.23 |

| 3: Transference |  |  |  |  |  |
| --- | --- | --- | --- | --- | --- |
| Analysis of Variance |  |  |  |  |  |
| Source | Adjusted Sum of Square | DF | Adjusted Mean Square | F-Value | Sig |
| Age: | 13.498 | 3 | 4.4994 | 10.38 | 0.000 |
| Gender | 1.223 | 1 | 1.2228 | 2.82 | 0.095 |
| Ethnicity | 2.823 | 1 | 2.8231 | 6.51 | 0.011 |
| Marital status | 3.866 | 2 | 1.9328 | 4.46 | 0.013 |
| Having children | 2.336 | 1 | 2.3364 | 5.39 | 0.021 |
| Seniority | 4.758 | 2 | 2.379 | 5.49 | 0.005 |
| Specialty | 5.431 | 4 | 1.3577 | 3.13 | 0.016 |
| Shift | 2.121 | 1 | 2.1207 | 4.89 | 0.028 |

**Support**

| 1: supervisor support |  |  |  |  |  |
| --- | --- | --- | --- | --- | --- |
| Analysis of Variance |  |  |  |  |  |
| Source | Adjusted Sum of Square | DF | Adjusted Mean Square | F-Value | Sig |
| Age: | 13.236 | 3 | 4.41196 | 9.54 | 0.000 |
| Gender | 0.042 | 1 | 0.0416 | 0.09 | 0.764 |
| Ethnicity | 0.152 | 1 | 0.15248 | 0.33 | 0.566 |
| Marital status | 3.341 | 2 | 1.67042 | 3.61 | 0.029 |
| Having children | 0.157 | 1 | 0.15654 | 0.34 | 0.561 |
| Seniority | 1.433 | 2 | 0.7163 | 1.55 | 0.215 |
| Specialty | 4.573 | 4 | 1.14328 | 2.47 | 0.045 |
| Shift | 1.888 | 1 | 1.88774 | 4.08 | 0.045 |

| 2: social/community support |  |  |  |  |  |
| --- | --- | --- | --- | --- | --- |
| Analysis of Variance |  |  |  |  |  |
| Source | Adjusted Sum of Square | DF | Adjusted Mean Square | F-Value | Sig |
| Age: | 6.2862 | 3 | 2.09538 | 5.79 | 0.001 |
| Gender | 0.3213 | 1 | 0.32125 | 0.89 | 0.347 |
| Ethnicity | 0.1811 | 1 | 0.18108 | 0.5 | 0.480 |
| Marital status | 2.7474 | 2 | 1.3737 | 3.8 | 0.024 |
| Having children | 0.0885 | 1 | 0.08853 | 0.24 | 0.621 |
| Seniority | 3.0956 | 2 | 1.54781 | 4.28 | 0.015 |
| Specialty | 4.722 | 4 | 1.18049 | 3.26 | 0.013 |
| Shift | 0.0555 | 1 | 0.05552 | 0.15 | 0.696 |

**Appendix 1- Section 2**

**ANOVA/ Demographic variables & Domains**

| 1: Stressors |  |  |  |  |  |
| --- | --- | --- | --- | --- | --- |
| Analysis of Variance |  |  |  |  |  |
| Source | Adjusted Sum of Square | DF | Adjusted Mean Square | F-Value | Sig |
| Age: | 7.501 | 3 | 2.5004 | 12.67 | 0.000 |
| Gender | 11.006 | 1 | 11.0056 | 55.79 | 0.000 |
| Ethnicity | 0.532 | 1 | 0.5321 | 2.7 | 0.102 |
| Marital status | 6.748 | 2 | 3.3738 | 17.1 | 0.000 |
| Having children | 0.017 | 1 | 0.0175 | 0.09 | 0.766 |
| Seniority | 14.469 | 2 | 7.2343 | 36.67 | 0.000 |
| Specialty | 9.145 | 4 | 2.2863 | 11.59 | 0.000 |
| Shift | 0.445 | 1 | 0.4449 | 2.26 | 0.135 |

| 2: Physio-psycho-social symptoms |  |  |  |  |  |
| --- | --- | --- | --- | --- | --- |
| Analysis of Variance |  |  |  |  |  |
| Source | Adjusted Sum of Square | DF | Adjusted Mean Square | F-Value | Sig |
| Age: | 11.076 | 3 | 3.6922 | 12.05 | 0.000 |
| Gender | 16.097 | 1 | 16.0974 | 52.55 | 0.000 |
| Ethnicity | 0.014 | 1 | 0.0141 | 0.05 | 0.83 |
| Marital status | 13.619 | 2 | 6.8093 | 22.23 | 0.000 |
| Having children | 0.776 | 1 | 0.7765 | 2.53 | 0.113 |
| Seniority | 12.538 | 2 | 6.2688 | 20.47 | 0.000 |
| Specialty | 11.438 | 4 | 2.8595 | 9.34 | 0.000 |
| Shift | 2.035 | 1 | 2.035 | 6.64 | 0.011 |

| 3: Coping |  |  |  |  |  |
| --- | --- | --- | --- | --- | --- |
| Analysis of Variance |  |  |  |  |  |
| Source | Adjusted Sum of Square | DF | Adjusted Mean Square | F-Value | Sig |
| Age: | 11.263 | 3 | 3.7544 | 12.99 | 0.00 |
| Gender | 20.707 | 1 | 20.7068 | 71.63 | 0.00 |
| Ethnicity | 3.254 | 1 | 3.2545 | 11.26 | 0.001 |
| Marital status | 2.961 | 2 | 1.4803 | 5.12 | 0.007 |
| Having children | 0.945 | 1 | 0.9449 | 3.27 | 0.072 |
| Seniority | 2.388 | 2 | 1.1942 | 4.13 | 0.017 |
| Specialty | 6.612 | 4 | 1.6529 | 5.72 | 0.000 |
| Shift | 1.353 | 1 | 1.3531 | 4.68 | 0.032 |

| 4: Social Support |  |  |  |  |  |
| --- | --- | --- | --- | --- | --- |
| Analysis of Variance |  |  |  |  |  |
| Source | Adjusted Sum of Square | DF | Adjusted Mean Square | F-Value | Sig |
| Age: | 6.1315 | 3 | 2.04385 | 10.59 | 0.000 |
| Gender | 0.1485 | 1 | 0.14852 | 0.77 | 0.381 |
| Ethnicity | 0.0003 | 1 | 0.00031 | 0.00 | 0.968 |
| Marital status | 1.7294 | 2 | 0.86472 | 4.48 | 0.012 |
| Having children | 0.0024 | 1 | 0.00241 | 0.01 | 0.911 |
| Seniority | 1.2447 | 2 | 0.62236 | 3.23 | 0.042 |
| Specialty | 1.8591 | 4 | 0.46477 | 2.41 | 0.05 |
| Shift | 0.3239 | 1 | 0.32395 | 1.68 | 0.196 |

**Appendix 1- Section 3**

**Regression**

| Stressors domain: |  |  |  |  |
| --- | --- | --- | --- | --- |
| Coefficients |  |  |  |  |
| Term | Coef | SE Coef | T-Value | P-Value |
| **Age:** |  | | | |
| 31-40 | 0.441 | 0.102 | 4.33 | 0.00 |
| 41-50 | -0.317 | 0.139 | -2.29 | 0.023 |
| > 50 | -0.07 | 0.105 | -0.66 | 0.509 |
| **Gender** |  | | | |
| Female | 0.966 | 0.129 | 7.47 | 0.00 |
| **Ethnicity** |  | | | |
| African | 0.183 | 0.111 | 1.64 | 0.102 |
| **Marital status** |  | | | |
| Divorced | -0.353 | 0.13 | -2.72 | 0.007 |
| Never married | 0.4147 | 0.0842 | 4.92 | 0.00 |
| **Having children** |  | | | |
| Yes | 0.033 | 0.11 | 0.3 | 0.766 |
| **Seniority** |  | | | |
| > 10 years of experience | 0.6097 | 0.0731 | 8.34 | 0.00 |
| > 15 years of experience | -0.142 | 0.127 | -1.12 | 0.265 |
| **Specialty** |  | | | |
| ICU nurse | 0.1455 | 0.0891 | 1.63 | 0.104 |
| OR nurse | -0.236 | 0.1 | -2.35 | 0.02 |
| ER burse | -0.292 | 0.115 | -2.54 | 0.012 |
| Other (labor and delivery (51), pediatric(52) | -0.552 | 0.107 | -5.14 | 0.00 |
| **Shift** |  | | | |
| Night | 0.1013 | 0.0674 | 1.5 | 0.135 |

| Physio-psycho-social symptoms: |  |  |  |  |
| --- | --- | --- | --- | --- |
| Coefficients |  |  |  |  |
| Term | Coef | SE Coef | T-Value | P-Value |
| **Age:** |  | | | |
| 31-40 | 0.561 | 0.127 | 4.43 | 0.00 |
| 41-50 | -0.363 | 0.173 | -2.1 | 0.037 |
| > 50 | 0.029 | 0.131 | 0.22 | 0.828 |
| **Gender** |  | | | |
| Female | 1.168 | 0.161 | 7.25 | 0.00 |
| **Ethnicity** |  | | | |
| African | 0.03 | 0.139 | 0.21 | 0.83 |
| **Marital status** |  | | | |
| Divorced | -1.066 | 0.162 | -6.6 | 0.00 |
| Never married | 0.041 | 0.105 | 0.4 | 0.693 |
| **Having children** |  | | | |
| Yes | 0.218 | 0.137 | 1.59 | 0.113 |
| **Seniority** |  | | | |
| Intermediate | 0.3534 | 0.0911 | 3.88 | 0.00 |
| Experienced | -0.742 | 0.159 | -4.68 | 0.00 |
| **Specialty** |  | | | |
| ICU nurse | -0.052 | 0.111 | -0.47 | 0.64 |
| OR nurse | -0.482 | 0.125 | -3.86 | 0.00 |
| ER burse | 0.011 | 0.143 | 0.08 | 0.938 |
| Other (labor and delivery (51), pediatric(52) | -0.614 | 0.134 | -4.59 | 0.00 |
| **Shift** |  | | | |
| Night | 0.2166 | 0.084 | 2.58 | 0.011 |

| Coping: |  |  |  |  |
| --- | --- | --- | --- | --- |
| Coefficients |  |  |  |  |
| Term | Coef | SE Coef | T-Value | P-Value |
| **Age:** |  | | | |
| 31-40 | 0.49 | 0.123 | 3.98 | 0.00 |
| 41-50 | -0.395 | 0.168 | -2.36 | 0.019 |
| > 50 | -0.236 | 0.128 | -1.85 | 0.066 |
| **Gender** |  | | | |
| Female | 1.325 | 0.157 | 8.46 | 0.00 |
| **Ethnicity** |  | | | |
| African | -0.453 | 0.135 | -3.36 | 0.001 |
| **Marital status** |  | | | |
| Divorced | -0.502 | 0.157 | -3.2 | 0.002 |
| Never married | -0.025 | 0.102 | -0.24 | 0.809 |
| **Having children** |  | | | |
| Yes | 0.241 | 0.133 | 1.81 | 0.072 |
| **Seniority** |  | | | |
| Intermediate | 0.2454 | 0.0885 | 2.77 | 0.006 |
| Experienced | -0.074 | 0.154 | -0.48 | 0.63 |
| **Specialty** |  | | | |
| ICU nurse | -0.035 | 0.108 | -0.33 | 0.745 |
| OR nurse | -0.286 | 0.121 | -2.35 | 0.019 |
| ER burse | -0.29 | 0.139 | -2.08 | 0.039 |
| Other (labor and delivery (51), pediatric(52) | -0.563 | 0.13 | -4.33 | 0.00 |
| **Shift** |  | | | |
| Night | -0.1766 | 0.0816 | -2.16 | 0.032 |

| Support: |  |  |  |  |
| --- | --- | --- | --- | --- |
| Coefficients |  |  |  |  |
| Term | Coef | SE Coef | T-Value | P-Value |
| **Age:** |  | | | |
| 31-40 | -0.367 | 0.101 | -3.65 | 0.00 |
| 41-50 | 0.087 | 0.137 | 0.63 | 0.528 |
| > 50 | 0.259 | 0.104 | 2.48 | 0.014 |
| **Gender** |  | | | |
| Female | 0.112 | 0.128 | 0.88 | 0.381 |
| **Ethnicity** |  | | | |
| African | 0.004 | 0.11 | 0.04 | 0.968 |
| **Marital status** |  | | | |
| Divorced | -0.058 | 0.128 | -0.45 | 0.651 |
| Never married | 0.2424 | 0.0833 | 2.91 | 0.004 |
| **Having children** |  | | | |
| Yes | 0.012 | 0.109 | 0.11 | 0.911 |
| **Seniority** |  | | | |
| Intermediate | -0.0011 | 0.0723 | -0.02 | 0.988 |
| Experienced | -0.318 | 0.126 | -2.53 | 0.012 |
| **Specialty** |  | | | |
| ICU nurse | 0.2482 | 0.0881 | 2.82 | 0.005 |
| OR nurse | 0.2124 | 0.0991 | 2.14 | 0.033 |
| ER burse | 0.085 | 0.114 | 0.75 | 0.457 |
| Other (labor and delivery (51), pediatric(52) | 0.062 | 0.106 | 0.58 | 0.562 |
| **Shift** |  | | | |
| Night | -0.0864 | 0.0667 | -1.3 | 0.196 |
