# Supplementary material for: Investigating Frontline Nurse Stress: Perceptions of Job Demands, Organizational Support, and Social Support During the Current COVID-19 Pandemic
Source: Front Public Health. 2022 May 25;10:839600. doi: 10.3389/fpubh.2022.839600 (PMC9204268; doi:10.3389/fpubh.2022.839600)
Supplement: Supplementary file 2 [file Data_Sheet_2.docx]

**Investigating Frontline Nurse Stress: Perceptions of Job Demands, Organizational Resources, and Social Support during the Current COVID-19 Pandemic**

**Questionnaire Instrument items**

| **Disclaimer** | COVID-19 is a highly contagious airborne virus that appeared in the US in January. The Centers for disease control recommend social distancing and face masks (KN95 and N95) to limit the spread of infection. The following survey will ask you questions related to your experience working with patients infected with COVID-19.    This is a survey study of front-line nurses working in HOSPITAL settings. This survey is being conducted to investigate and help us understand the significant sources of stressors nurses face during the COVID-19 pandemic and explore the coping strategies employed by the nursing staff to overcome this stress. This survey should take approximately 10 minutes to complete, and your participation is voluntary. If you decided to fill out the survey, you could choose to provide an address to receive a $20 gift card, to provide VENMO ID for cash compensation, or to donate the amount to a food bank. No identifiable information will be collected- If you decided to be compensated, your information will be kept confidential and will be directly deleted from the system immediately after transferring the compensation.    If you have questions about this study, you may contact the study's lead investigator.    As a research participant, if you have questions about your rights, you may contact to the XXX University, Office of Research Compliance, or the Institutional Review Board by phone XXX or email at IRBadmin@XX or IRBChair@XXX. |
| --- | --- |
| **Demographic Factors** | **Age: (1)** <30 **(2)** 31-40 **(3)** 41-50 **(4)** >50 |
|  | **Gender: (1)** Male **(2)** Female |
|  | **Ethnicity: (1)** White **(2)** African American **(3)** American Indian or Alaskan Native **(4)** Asian **(5)** Native Hawaiian or Pacific Islander **(6)** Hispanic |
|  | **Marital Status: (1)** Married **(2)** Divorced **(3)** Never Married |
|  | **Do you have children? (1)** No **(2)** Yes |
|  | **Seniority (1)** Beginner (less than 5 years of job experience) **(2)** Intermediate (6-10 years of job experience) **(3)** Advanced (over 10 years of job experience) |
|  | **Specialty: (1)** Head nurse, **(2)** general nurse, **(3)** ICU, **(4)** OR **(5)** ER, **(6) Other:** Please specify |
|  | **Shift type: (1)** Day shift (primarily); **(2)** Night shift (primarily) |
| **Stressors** | **Please describe your feelings during this crisis – (1)** Strongly Disagree, **(2)** Agree, **(3)** Neither Agree nor Disagree, **(4)** Agree, **(5)** Strongly Agree |
| **Taking care of patients related stress** | I am worried about my infected patients getting worse or dying in front of me. |
|  | I am worried about communication with my patients and their families. |
|  | I am worried about my ability to provide the best care to my patients. |
| **Assignment and workload related stress** | I feel more stress now than I did before the COVID-19 pandemic. |
|  | I am worried about accidentally spreading the infection to my friends and family. |
|  | I am worried that I will have to take care of my infected colleagues. |
|  | I am worried about receiving additional infected patients or suspected cases. |
|  | I am worried about keeping a face mask on for a long period of time. |
|  | I am worried about being assigned to a front-line job with COVID-19 patients. |
| **Colleagues, staff, and personal life related stress** | I am worried about becoming infected. |
|  | I am worried about friends, neighbors, and colleagues trying to avoid me during the crisis. |
|  | I am worried about my colleagues becoming infected. |
|  | I am worried about my family becoming infected. |
|  | I get worried when I see that my colleagues are worried. |
| **Lack of knowledge about Covid-19 related stress** | I am worried about the lack of specific treatment for COVID-19. |
|  | I am worried about a lack of psychological support for patients. |
|  | I am worried about a lack of guidance on how to deal with COVID-19 patients. |
|  | I am worried about the constant changes to regulations related to COVID-19. |
|  | I am worried about the lack of a vaccine for COVID-19. |
|  | I am worried about the stay-at-home order being lifted too early. |
|  | I am worried about how long the COVID-19 pandemic will last. |
|  | I am worried about hearing the news/reports for the number of new cases every day. |
|  | I am worried about being constantly screened for infection. |
| **Environmental related stress** | I am worried about the lack of support in my organization, such as psychological materials and resources. |
|  | I get worried when I receive news related to hospital staff shortages. |
|  | I am worried about my stress levels as a health factor. |
|  | I get worried when I hear about PPE shortages. |
|  | I get worried when I hear about ventilators shortages. |
|  | I get worried when I hear about face mask shortages. |
|  | **Please describe your feelings during this crisis – (1)** Strongly Disagree, **(2)** Agree, **(3)** Neither Agree nor Disagree, **(4)** Agree, **(5)** Strongly Agree |
| **Nurses Feelings** | I feel that my major front-line job stressors come from heightened social and professional responsibilities. |
|  | I feel nervous or frightened during my work shift. |
|  | I would be upset if I had to work overtime during the crisis. |
|  | I try to reduce exposure to COVID-19 patients. |
|  | I have thought about leaving my job in the last 3 months. |
|  | I think about leaving my job more as the infection rate increases. |
|  | I feel angry/mad because my workload is greater and more dangerous than others, such as doctors, technicians. |
|  | I often think of calling in sick. |
|  | I feel I should receive bonus compensation during or after the pandemic. |
|  | I feel I would benefit from psychological care. |
|  | I feel I would benefit from grief counseling. |
|  | I feel I would benefit from counseling for trauma. |
|  | I think I would benefit from family counseling. |
|  | Please, describe your negative emotion during this crisis. Example: fatigue, discomfort, fear, anxiety, etc. |
| **Coping strategies** | **Please indicate to which degree you use the coping mechanism – (1)** Strongly Disagree, **(2)** Agree**, (3)** Neither Agree nor Disagree**, (4)** Agree, **(5)** Strongly Agree |
|  | **Avoidance:** |
|  | Avoiding working overtime to reduce exposure to COVID-19 patients. |
|  | Avoiding working with COVID-19 patients (working back-line). |
|  | Avoiding media news about COVID-19 and related infection and mortality rates. |
|  | Avoided being with people in general. |
|  | **Problem solving:** |
|  | Following strict protective measures (handwashing, face masks, protective gear, etc.) |
|  | Learning about COVID-19, its prevention, and mechanism of transmission. |
|  | Avoiding circumstances where I can get infected, such as avoiding transportation, crowded places, etc. |
|  | Taking the necessary precautions when grocery shopping. |
|  | **Transference:** |
|  | Practicing religious or spiritual activities such as prayer, meditation, and reading. |
|  | Practicing relaxation activities in your free time, such as watching movies, reading, exercising, etc. |
|  | Chatting with family and friends to relieve stress and obtain support. |
|  | Seeking help from a psychologist. |
| **Support measures** | **Please describe your feelings during this crisis – (1)** Strongly Disagree, **(2)** Agree, **(3)** Neither Agree nor Disagree**, (4)** Agree, **(5)** Strongly Agree |
| **Supervisor support** | My supervisor cares about my opinions. |
|  | My supervisor cares about my well-being. |
|  | My supervisor strongly considers my goals and values. |
|  | My supervisor shows very little concerns for me. |
| **Social/ community support** | **Please state whether you experienced the following social event within the last 3 months:** (**0)** No (**1)** Yes |
|  | A family member or a friend was physically there for you in a stressful situation. |
|  | A family member or a friend provided you with a place where you could get away for a while. |
|  | A family member or a friend watched after your home when you were away (children, pets, plants, light cleaning, etc.). |
|  | A family member or a friend did some activity together to help you relax. |
|  | A family member or a friend told you that you are OK, and everything will be fine. |
|  | A family member or a friend lets you know that he/she will always be around if you need assistance. |
|  | A family member or a friend offered or provided transportation assistance. |
|  | A family member or a friend listened to you talking about your private feelings. |
|  | A family member or a friend expressed interest and concern in your well-being. |
|  | A family member or a friend joked and kidded to try to cheer you up. |
